# Asthma and air pollution

**DOI:** 10.1186/1824-7288-40-S1-A75

**Published:** 2014-08-11

**Authors:** Giuliana Ferrante, Roberta Antona, Velia Malizia, Laura Montalbano, Stefania La Grutta

**Affiliations:** 1 Dipartimento di Scienze per la Promozione della Salute e Materno-Infantile “G. D’Alessandro”, Università degli Studi di Palermo, Palermo, 90133, Italy; 2 Istituto di Biomedicina e Immunologia Molecolare- IBIM “A. Monroy”, Consiglio Nazionale delle Ricerche, Palermo, 90146, Italy

## 

During the last decades research all over the world has highlighted the deleterious effects of pollution on respiratory health of adults and children. Nevertheless, air pollution still represents a significant threat to health. Children are more sensitive than adults to pollutants for several factors: increased respiration relative to body size; physiologic immaturity of respiratory and immunologic systems; low metabolic capacity; longer life expectancy.

Several studies demonstrated an association between exposure to outdoor pollutants and respiratory diseases in childhood. Outdoor pollutants, such as nitrogen oxides (NOx), particulate (PM), carbon monoxide (CO), carbon dioxide (CO_2_), ozone (O_3_), sulfur dioxide (SO_2_), may provoke cytotoxic and functional damages in the airways through oxidative stress and inflammation. During the last decades the amount of pollutants from vehicular traffic has significantly increased, especially in urban areas. Recent epidemiological studies have shown that vehicular traffic represents the main source of outdoor pollutants and that it may increase the risk of respiratory outcomes (cough, phlegm, wheeze, asthma) in children through short-term and long-term effects on airways, lung function and allergic sensitization [1].

Indoor pollution is also particularly dangerous, mainly for children and adolescents, that typically spend most of the time in confined spaces (home, school and public spaces). Indoor pollutants concentration depends on external environmental pollutants filtered inside buildings, pollutants generated inside buildings (domestic work) and pollutants generated by personal activities. Combustion products (tobacco smoke and wood burning), CO, CO2, volatile organic compounds (VOC), microbial agents (fungi and bacterial endotoxins), organic products (pet derived and mite allergens, dampness, mold derived components) are the most important indoor pollutants. There is growing epidemiological evidence that indoor allergen exposure may contribute to the development of allergic respiratory symptoms, such wheezing, coughing and asthma in children [2].

Tobacco smoke is one of the environmental pollutants influencing morbidity and death rate in childhood as it is responsible for adverse health effects in both prenatal and postnatal life. Homes remain a site where children are dangerously exposed to environmental tobacco smoke (ETS). The combination of tobacco smoke pollutants which remain in an indoor environment, the so-called ‘*third-hand smoke*’ (THS), represents a new concept in the field of tobacco control [3].

Children still need to be protected with strict air quality standards, in order to improve their respiratory health. Therefore, policies that ensure better air quality are strongly desirable all over the world.
